# Supplementary material for: WordCommentsAnalyzer: A windows software tool for qualitative research
Source: F1000Res. 2018 Sep 4;7:536. Originally published 2018 May 3. [Version 2] doi: 10.12688/f1000research.14819.2 (PMC6137411; doi:10.12688/f1000research.14819.2)
Supplement: Supplementary file 3 [file f1000research-7-17614-s0002.tgz › b2a79496-4ada-47c4-9682-a99ce867836d.zip!/Eight Iranian Health Journals/Journal of Research in Rehabilitation Sciences _0.docx]

© Journal of Research in Rehabilitation Sciences . This work is licensed under CC BY-NC 4.0 (https://creativecommons.org/licenses/by-nc/4.0/).

Volume & Issue: دوره 13، شماره 5: 1396 (در حال تکمیل)

Title: تأثیر خستگی عضلات ارکتور اسپاین بر حس وضعیت تنه، ران و زانوی مردان کاراته‌کار

http://jrrs.mui.ac.ir/index.php/jrrs/article/view/2910

مقدمه: حس عمقی و تعادل، نقش قابل ‌توجهی در ارتقای عملکرد و پیشگیری از آسیب‌های حاد و مزمن ورزشی ایفا می‌کند. خستگی عضلانی می‌تواند یکی از عوامل ایجاد اختلال در رسیدن اطلاعات از منابع حسی و اندام‌ها به مغز باشد و تعادل را تحت تأثیر قرار دهد و تأثیر خستگی عضلانی بر این حس کمتر مطالعه شده است. هدف از انجام پژوهش حاضر، بررسی تأثیر خستگی عضلات ارکتور اسپاین بر حس وضعیت تنه، ران و زانوی کاراته‌کاران مرد بود. مواد و روش‌ها: 10 مرد کاراته‌کار حاضر در مسابقات لیگ کشوری، در این مطالعه شرکت نمودند. خطای مطلق بازسازی زوایای فعال تنه در 45 درجه فلکشن، ران در 30 درجه ابداکشن و فلکشن زنجیره حرکتی باز و زانو در زوایای 30 و 60 درجه فلکشن زنجیره حرکتی بسته به ‌صورت اندازه‌گیری قبل و بعد با کمک فیلمبرداری محاسبه گردید و در نرم‌افزار AutoCAD مورد تجزیه و تحلیل قرار گرفت. خستگی عضلات ارکتور اسپاین قبل و پس از خستگی با استفاده از آزمون Sorensen اندازه‌گیری شد. یافته‌ها: بر اساس آزمون Paired t، خستگی عضلات ارکتور اسپاین تأثیر معنی‌داری بر حس وضعیت تنه در فلکشن 45 درجه (046/0 = P) و مفصل ران پای غالب (001/0 = P) و غیر غالب (001/0 = P) در حرکت ابداکشن 30 درجه داشت. خستگی، تأثیر معنی‌داری را بر حس وضعیت مفصل ران پای غالب (434/0 = P) و غیر غالب (703/0 = P) در حرکت فلکشن 30 درجه، وضعیت مفصل زانوی پای غالب (148/0 = P) و غیر غالب (204/0 = P) در حرکت فلکشن 30 درجه و پای غالب (417/0 = P) و غیر غالب (439/0 = P) در زاویه 60 درجه نشان نداد. نتیجه‌گیری: خستگی عضلات ارکتور اسپاین در کاراته‌کاران، باعث به وجود آمدن اختلال حس وضعیت تنه و ران می‌گردد که می‌تواند عامل احتمالی ظهور آسیب در اندام تحتانی و بی‌ثباتی به ویژه در ران باشد. با این ‌حال، ممکن است خستگی عضلات ارکتور اسپاین تأثیر معنی‌داری بر حس وضعیت زانو نداشته باشد و بر پتانسیل آسیب این مفصل تأثیر نگذارد.

Title: پاسخ فیزیولوژیکی استرس اکسیداتیو به ریکاوری آب سرد به دنبال فعالیت سرعتی شدید در مردان تمرین‌ کرده

http://jrrs.mui.ac.ir/index.php/jrrs/article/view/2935

مقدمه: هدف از انجام مطالعه حاضر، بررسی میزان تغییرات برخی شاخص‌های استرس اکسیداتیو مانند پروتئین کربونیل (Protein carbonyls یا PC) و مالون دی‌آلدئید (Malondialdehyde یا MDA) پس از فعالیت سرعتی تکراری (Repeated sprint ability یا RSA) و پس از آن غوطه‌وری در آب سرد (Cold water immersion یا CWI) بود. مواد و روش‌ها: 20 ورزشکار تمرین‌کرده برای شرکت در این پژوهش انتخاب شدند. پس از انجام RSA، 10 نفر از آزمودنی‌ها داخل آب سرد با دمای 14 درجه سانتی‌گراد رفتند و 10 نفر دیگر در دمای اتاق به شکل غیر فعال روی صندلی نشستند. خونگیری قبل و پس از انجام RSA، پس از ریکاوری در آب سرد و همچنین، 24 ساعت پس از آخرین خونگیری انجام گرفت. یافته‌ها: انجام RSA منجر به افزایش مقادیر سرمی PC (001/0 = P، 97/3 = t) و MDA (001/0 = P، 54/9- = t) شد. CWI پس از RSA تأثیر معنی‌داری بر مقادیر سرمی PC داشت. اختلاف میانگین‌ها کاهش معنی‌دار بلافاصله پس از ریکاوری و 24 ساعت پس از ریکاوری نسبت به قبل از ریکاوری و همچنین، 24 ساعت پس از ریکاوری نسبت به بلافاصله پس از آن را نشان داد. یافته‌های مربوط به آزمون Repeated measures ANOVA روی مراحل اندازه‌گیری نشان داد که تنها اثر اصلی مراحل اندازه‌گیری معنی‌دار بود (001/0 = P)، اما اثر اصلی گروه (572/0 = P) و تعامل مراحل اندازه‌گیری با گروه (915/0 = P) معنی‌دار نبود.نتیجه‌گیری: انجام فعالیت شدید متناوب، منجر به افزایش استرس اکسیداتیو می‌شود، اما CWI در مقایسه با قرارگیری در دمای اتاق، تأثیرات سودمندتری ندارد.
